# Perceptions of death, dying and the body in Vietnamese culture: A qualitative exploration from a study examining the potentials of minimally invasive tissue sampling in Vietnam

**DOI:** 10.1016/j.ssmqr.2026.100714

**Published:** 2026-06

**Authors:** Nhung Phuong Doan, My Nguyen Le Thao, An Luu Phuoc, Alix Day, Halina Suwalowska, Ho Dang Trung Nghia, Mary Chambers, Ta Thi Dieu Ngan, H. Rogier van Doorn, Jennifer Ilo Van Nuil

**Affiliations:** a Oxford University Clinical Research Unit, Hanoi and Ho Chi Minh City, Viet Nam; b Ethox Centre, Nuffield Department of Population Health, University of Oxford, Oxford, UK; c Hospital for Tropical Diseases, Ho Chi Minh City, Viet Nam; d Nuffield Department of Medicine, University of Oxford, Oxford, UK; e National Hospital for Tropical Diseases, Hanoi, Viet Nam

**Keywords:** Death, Dying, Minimally invasive tissue sampling, Vietnam, Qualitative

## Abstract

**Background:**

Perceptions of death and dying has been attracting more attention in recent years with more advocacy on improving the end-of-life experiences and providing more support for families during these times. While these topics are important, Western views about death and dying have been more dominant, leaving a knowledge gap in how death and dying are perceived in Asian cultures. We used qualitative methods to explore how death and dying was perceived in Vietnamese culture, and examine the implications for related practices, including minimally invasive tissue sampling.

**Methods:**

We conducted 59 in-depth interviews and five focus group discussions with key informants, healthcare professionals, community stakeholders and relatives of recently deceased people. We used a form of thematic analysis and explored the perceptions of death and dying, with particular focus on the concepts of a good death and the meaning of the body in Vietnamese culture.

**Results:**

Perceptions and attitudes surrounding death and dying varied, from an acceptance attitude to a fearful and anxious view when talking about death. A good death was usually perceived as dying at home and surrounded by family, with no worries or regrets for both the deceased and the family, and keeping an intact body. The body has a sacred meaning to the family that is handled with great care and respect.

**Conclusion:**

Death and dying in Vietnamese culture is a communal affair with a considerable role of the family on the dying experience of the person. Any related practices, such as end-of-life care or minimally invasive tissue sampling, should consider the cultural aspects to improve its acceptability.

## 1 Introduction

Death and mortality have been a focus of humanity, and thought about through spirituality, art, philosophy, and science from before the beginning of written history. Prior to the written records, archaeologists have argued that beliefs regarding the importance of death and the potential for life after death have existed since Palaeolithic times or perhaps before via burial grounds and artefacts within the burial sites (Hovers & Belfer‐Cohen, 2018). Perceptions of what death means have changed over time and often vary greatly between cultures. In pre-modern to early modern eras when spiritual beliefs, e.g. animism, and religious institutions first became embedded in societies, religion provided communities with explanations and meaning about death. Aspects of religion influenced people to accept their mortality as a normal part of life, regardless of what would happen after death, according to their belief systems (Arora, 2021).

During the late 18th to 20th century, sciences, particularly medical sciences, offered alternative perspectives on death and attitudes started to change where *“death became a morbid reality that threatens life”* (Arora, 2021, p. 348). In other words, death was not accepted as a part of life but something that was perceived as a threat to push off or delay. Death, at this time, also became medicalised where biological death was not always the same as cultural death, that is, the point at which someone had undergone all the rituals required in that culture. Biological death was typically pronounced by medical professionals changing the meaning of life and death (Lock & Nguyen, 2018). Another paradigm shift was argued to have occurred in the second half of the 20th century, where those dying claimed more agency in their death and associated rituals (Arora, 2021). Although not universal, ethnographers more recently have studied the agency in ‘scripting’ or ‘choreographing’ one's death, for example in Thailand in the context of Buddhist rituals entangled with the emergence and use of biotechnologies, e.g. ICU care, for end of life care (S. Stonington, 2020).

Scholars have proposed a spectrum of perceptions related to death and mortality. For example, death is perceived as a normal occurrence to be accepted, or death is something to be feared and denied or, as is often the case, death is perceived as a combination of complex ideas existing somewhere in between these two views (Arora, 2021). Anthropological conceptualisations of death similarly frame it on two extremes: the ways in which social life persists (after biological death) via social rituals with an emphasis on striving for a ‘good death’ and on the other hand, the perceptions related to the physical body (or corpse) (Engelke, 2019). Indeed, death is often perceived as a mixture of these extremes, depending on the specific context. Medical technologies and the advancement of hospital units (e.g. intensive care units) complicated the boundaries within biological death, for example, how we define ‘death’ if a person can be pronounced brain dead while being connected to a mechanical ventilator that artificially keeps their (other) organs viable (Kaufman, 2020; Lock & Nguyen, 2018). Across this complex and fluid backdrop, it is clear that there are multiple and everchanging perceptions of how death is conceptualized among and between cultures.

As part of a formative study examining the acceptability of minimally invasive tissue sampling (MITS) and exploring suggestions for its potential implementation in Vietnam, it was critical to understand the myriad beliefs about death and dying in this context. We published findings discussing the hypothetical acceptability of MITS in Vietnam [Blinded ref for review], however, the topics of death and dying became a full analysis on its own. We aimed to understand the perceptions and attitudes regarding social death, as well as how people perceived the body after death, both biologically and culturally. In this article, we examined data surrounding the perceptions of death, dying, and the body in Vietnamese culture. As the context of the study was MITS, we also sought to understand how these perceptions could impact participants’ views about MITS and have implications for other relevant topics, such as end-of-life care.

### 1.1 The context of palliative care in Vietnam

The Vietnamese Ministry of Health issued a national palliative care guideline in 2006 focused on those living with cancer and HIV. However to date, the majority of those needing palliative care remain at home with hospital-based care available mostly at hospitals in major cities, like Ho Chi Minh city and Hanoi (Krakauer et al., 2018). There have been a few initiatives to expand palliative care services in Vietnam. For example, the Department of Palliative Care at the Ho Chi Minh city Oncology Hospital developed a very limited integrated hospital and home-based program in 2011 but the home-based care services were not covered by national insurance so families had to pay out of pocket (V. Nguyen et al., 2023). The first pediatric palliative care unit was implemented in one of the major children's hospitals in 2017 but these services remained hospital-based, while most of the children passed away in the home leaving a gap in care (Adelmann et al., 2025). For families who can pay out of pocket, there are specialised care professionals who could provide home-based care but for the majority of the Vietnamese population, the family becomes the ones who care for those who are at the end of life.

### 1.2 Vietnamese context of death and dying

There are 54 recognised ethnic groups and various religious practices in Vietnam and as such, there are multiple views on death and dying. Vietnamese “folk religious beliefs” (“*tín ngưỡng dân gian Việt Nam”)* is followed by 86 % of citizens (General Statistics Office of Vietnam, 2020) and is a decentralised syncretism of Taoism, Buddhism, and Confucianism which underpins worship of the dead, ancestors, and “ghosts” (Hung & Lam, 2024). The remaining 14 % of the population follow other religions such as Catholicism, Christianity, Islam, animism, or atheists, each with a different approach to death and dying (Hung & Lam, 2024). One example are the Vietnamese Caodaists (*Cao Đài* religion), a southern autochthonous religion of around 3 million followers. Within this religion, human “completeness” comprises the “three bodies”: the physical (“*đê hút xác than”*), the spiritual or ghostlike (“true mind”, “*hồn ma*”), and the amorphous true spirit (“soul”, “*linh hồn*”), with corresponding burial rituals (Jammes & Shuai, 2020). The Cham communities, whose religion is based on either Islam or Hindi, total around 179,000 (General Statistics Office of Vietnam, 2020). The Cham Ahier (following Hindu beliefs) conduct cremation or burial based on familial lineages, while burials in “cremation lineages” are reserved for “bad deaths”. Cremations often involve removal of pieces of frontal bone (*talang*) as tokens for passage to the afterlife (Linh, 2022).

Despite these differences, dying at home is considered by most to be essential for a good death, and family are expected to surround the dying person, providing “warmth” for the spirit's reincarnation (L. T. Nguyen et al., 2014). Dying outside the home risks the dead's spirit becoming a wandering soul (“*hồn ma lang thang*”), thus in-hospital deaths are avoided (S. Nguyen & Giap, 2019). Oncology nurses in contact with dead bodies also report facing stigma over concerns of their capacity to “taint” the living, especially children (T. Nguyen et al., 2017). After a home-death, monks or capable elders are called to chant to encourage spiritual transition to the metaphysical world. Burial is conducted between two to seven days later, and the official morning period is 100 days (S. Nguyen & Giap, 2019). Despite burial culture's embeddedness, cremation is also increasing due to urbanisation, reaching 75 % and 65 % in Ho Chi Minh City and Ha Noi (Chau, 2020), and “*houses of remains*” (“*nhà lưu tro cốt*”, “ash houses”) having risen from 7 (1954-75) to 204 (2017-24) among Catholic communities in HCMC (D. T. Phuong, 2024).

## 2 Methods

This research was conducted by researchers from the Oxford University Clinical Research Unit – Vietnam (OUCRU) in collaboration with long-term partners across Vietnam who we have collaborated in various projects. Our partners included medical universities, provincial Centres for Disease Control and Prevention (CDCs), National Hospital for Tropical Diseases, and Ho Chi Minh City Hospital for Tropical Diseases. Data collection started in October 2021 and ended in May 2023. We conducted the study in Hanoi, Ho Chi Minh city (HCMC), and Dak Lak province^2^ to capture diverse perspectives across various geographical locations in the country.

### 2.1 Participants and recruitment

To explore distinctive local cultures, we also aimed to recruit participants from both rural and urban areas, and people from ethnic minority groups. Participants included four main groups: key informants, including public health experts, researchers, and medical legislative officers; healthcare workers, including doctors and nurses from ICUs, emergency departments and general wards, forensic doctors, and pathologists; community stakeholders, including community leaders, religious leaders, local authorities; and relatives of recently deceased individuals. To avoid evoking painful emotions, we only recruited people who had bereavement experience more than six months ago. We recruited the initial participants through existing networks of our study partners. For example, medical universities and hospitals helped recruit healthcare workers and medical students, while our Public Engagement team at OUCRU and local CDCs assisted in recruiting potential community stakeholders. Snowball sampling was also employed for participants to identify potential people who might provide insight for the study. Local officers, including healthcare workers and administrative officers, also assisted in contacting stakeholders and family members. Considering the sensitivity of the research topic, we tried to engage with potential participants through trustworthy gatekeepers from our networks and made sure we explained carefully and thoughtfully the purpose of the study during recruitment. We also invited family members to invite someone else to sit with them during the interview for emotional support.

### 2.2 Data collection

We collected data using in-depth interviews (IDIs) and focus group discussions (FGDs). Semi-structured topic guides (Appendix 1) were used and revised throughout the study. All participants were compensated for their time in cash following local study payment norms. Data were collected by three experienced social science researchers (NPD, MNLT, ALP) in Vietnamese, with regular team meetings to discuss findings and impressions from the interviews as they were being conducted. We audio recorded the IDIs and FGDs, transcribed the audio verbatim into language spoken, anonymised the transcript, and used Nvivo 12 to organise the analysis process. There was an exception of two IDIs where the participants did not give consent to audio recording. The researchers then took detailed notes to ensure the consistency of data analysis. To ensure that the interpretation was agreed within the team, which included non-Vietnamese speakers, the Vietnamese researchers discussed and selected 12 transcripts, which were then translated to English by contracted translators. The selected transcripts were those that demonstrated the main themes across the dataset. After translation, the transcripts were also checked by the research team before analysis. Participant privacy was ensured throughout the study by storing personal information and the dataset in a project drive that could only be accessed by research team members. Raw transcripts were de-identified before analysis and deleted after deidentification.

In total, we conducted 59 IDIs, within which one was a paired interview. We also conducted five FGDs across the study sites, with five to ten participants in each FGD. The IDIs lasted from 17 to 167 min, while the FGDs were on average 2 h and 30 min each.

### 2.3 Data analysis

We used a form of thematic analysis (Braun & Clarke, 2022) to guide this analysis. Data familiarisation began during regular debrief sessions with the study team and all transcripts were read carefully by the three social science researchers (NPD, MNLT, ALP) prior to initiating the coding process. The initial coding was done by NPD and ALP. As this analysis was focused on specific topics, including perceptions of death, dying and the body, we used a combination of deductive and inductive coding to develop the codes and thematic maps. The closed coding contained semantic codes related to the topics of inquiry, e.g. perceptions of the body, while the open codes tended to be more latent, e.g. the body as sacred. We grouped and regrouped the subcodes and named the sub-themes, and discussed with Vietnamese co-investigators and experts who were well versed in the topics to improve the confirmability of findings. The results presented below are organised by the two main categories, with sub-themes described in more detail.

### 2.4 Ethics approvals

The study received ethics approval from Oxford University Tropical Research Ethics Committee (OxTREC), and the review boards of the National Hospital for Tropical Diseases, Hanoi; the Hospital for Tropical Diseases, Ho Chi Minh city; and the provincial Centres for Disease Control in Dak Lak province. All participants provided written informed consent prior to participating in the study.

## 3 Results

Of 97 potential participants contacted, 96 agreed to participate (Table 1). Most participants were living in Hanoi and Ho Chi Minh city and around 20 % were from Dak Lak. In terms of participant groups, half of the participants were healthcare workers. We also recruited a relatively similar number of participants from the general public. Despite efforts, we only could interview two people from Ede ethnic group due to langague barriers as well as the shorter fieldwork period for the study site in Dak Lak. We did not collect information about gender of the participants since the aim of the study was to examine different perceptions of death and dying in general and across different ethnic groups.

**Table 1:** Demographic details of study participants.

	n (%)
Study sites	
Hanoi	41 (42.71)
Ho Chi Minh city	36 (37.50)
Dak Lak	19 (19.70)
Participant groups	
Key informants	8 (8.33)
Healthcare workers	48 (50.00)
Community stakeholders	28 (29.17)
Family members	12 (12.50)
Ethnic groups	
Viet	94 (97.92)
Ede	2 (2.08)
Total	**96 (100)**

First, we discuss the perceptions of death and dying from the participants' point of view including how death is a universal event but perceptions about the afterlife were specific to different groups. We also discuss what is meant by a *‘good death.’* In the second part, we discuss the perceptions and meaning of the physical body: the body as sacred as opposed to the body as temporary. We will also link these results to perceptions of MITS where appropriate.

### 3.1 Perceptions of death and dying

#### 3.1.1 Fate, the afterlife, and acceptance

The perceptions surrounding death varied across the narratives of the participants. First, death was described as a process, not one moment in time (i.e. biological death) but included the moments before death until after the death rituals finished. One common perception was that death is inevitable. This was a universal truth that all living beings face. Death was discussed as a natural progression that would eventually happen in every person's life. *“Dust will eventually return to dust” –* this perception was deeply rooted in some people's understanding of the life cycle as in a song called “Cát bụi” (Dust) composed by a famous Vietnamese artist Trinh Cong Son, which was released in 1974: “Hạt bụi nào hoá kiếp thân tôi/Để một mai tôi về làm cát bụi” (Trinh Cong Son Foundation, 2025). This can be translated roughly as “What specks of dust transformed into me/Then one day I will return to dust”.

*Well, death, what do I think it is? It is a loss, but sometimes this loss, what can it be? It is unpredictable and unavoidable. And, sometimes people call it fate. (A key informant in Dak Lak)*

Another way death was described was as something that has already been written in an individual's fate. The moment of death had already been inscribed in their destiny; it is unchangeable. And when that time comes, people have no choice but to embrace it. Viewing death as a natural course of life could make people more likely to accept it.

*Well, like my father's death, it was a law of nature, of life. Birth, aging, sickness and death. (A family member in Hanoi)*

Although the idea of death as an event was universal, further exploration led to various perceptions surrounding what would happen after death. For some, death represented the transition to a state of peace where a person would no longer suffer from physical or emotional pain. Death marked the termination of one's earthly journey, including the associated pain and suffering.

*[My dad’s] death is a release to him. Because it had reached to the [end] point so it had to be that way. (A family member in Ho Chi Minh city)*

For some, this meant that death was the end point for an individual and nothing else would happen beyond that moment.

*For example, some people, who have no religion, don’t believe in saints or gods. They don’t believe in the afterlife, either. These people then say death is the end. Death means everything’s over. After death, there’s nothing. (A community stakeholder in Ho Chi Minh city)*

In some religions or cultures, death is not the end of life but rather a commencement of a new cycle where a person would rebirth into a different life and start a new beginning. This excerpt below belongs to a Buddhist monk who talked about death according to a Buddist perspective.

*According to the Buddhist point of view, when a person dies, it is not the end. But it is the beginning of a new life. That is, when a person is born, it is the beginning of something … and preparing for an end. And when a person dies, they prepare for another life. It’s repetitive. That means death is not the end. It is a cyclical cycle. (A community stakeholder in Hanoi)*

The excerpts above do not just illustrate how people perceived death and an afterlife, it also indicates attitudes surrounding acceptance toward death. Accepting death and preparing for it was how some people choose to face death. The preparation was not just for the dying person, but it also gave families time to be emotionally ready when death came. Accepting that death was inevitable did not always mean that talking about it came easily. For some, talking helped prepare for death.

*In my opinion, just talk about [death] directly. Because it's true, it could happen to anyone. We just follow that. I will mentally prepare, I’ll arrange everything. That way, it's still better than leaving [unsolved] things behind … If I leave suddenly my loved ones who are left behind would have to deal with a lot of things that might not go according to my wishes. So, I choose to do … whatever is best for my loved ones. (A family member in HCMC)*

However, not all people calmly accepted and prepared for the death. For some people, death was a fearful event that made people anxious. Some people even tried not to talk about death for fear of bad luck.

*Well, of course, every man has to be afraid standing in front of death. Everyone fears death. (A community stakeholder in HCMC)*

#### 3.1.2 A “good death”- “yên nghỉ”

When talking about death (if any), Vietamese people usually use the terms *“yên nghỉ”* or *“ra đi thanh thản”,* which then is back translated to English as a restful death or resting in peace. While Vietnamese language does not have a specific term that could be literally translated as “a good death” in English, the relatively equivalent Vietnamese term for *“a good death”* shows how the perceptions of Vietnamese people surrounding death were shaped; that is, death should be peaceful and restful. This included several components.

##### 3.1.2.1 A good death is being beside your loved ones at home

Spending their last moments with loved ones is considered the wish of a dying person. From the narratives of our participants as exemplified by the quote below, “*a good death”* meant dying at home and being surrounded by their loved ones. Home was also a place where they felt safe more generally and free from pain during the dying process. We chose this excerpt from a healthcare worker to further illustrate that there was no difference in how *“a good death”* was conceptualized either by the general public or by health professionals.

*In most cases, you know, in our Vietnamese culture, the dying wishes to return to their home, where it is familiar to them, where they can have their loved ones by their side, although they are unconscious and they can’t talk. They can feel the warmth, there’s no fear there, you know. Because they can’t live any longer, they just want to see the people they love and to have them by their side. (A healthcare worker in HCMC)*

The last moments of a person were also important to the family. The family will gather around to take care of their loved ones and make sure that there was at least one member beside the dying person at all times. Family members who lived far away would be informed so that they could make arrangements to return home and spend the last days with their loved ones. This was not only true for participants from the Kinh majority people, but also from the Ede ethnic minority. In this excerpt below, a key informant based in a rural commune in Dak Lak shared his understanding about Ede's culture as an administrative officer working with the ethnic group.

*I: So, for Ede people, they also want to be back to their home before they die …*

*P: That’s right. After returning from the hospital, they will cook some meals for family members who returns from far away can be together and eat. It’s for the dying person to be able to meet all the family before they die. (A key informant in Dak Lak)*

Dying at home also had a spiritual importance beyond its emotional meanings to both the deceased and the family. Many participants mentioned a common tradition that a person dying outside of their home would become a wandering spirit, one who had no place to rest (Bodemer, 2005; Son & Nga, 2019). While *“yên nghỉ”* or a restful death was what Vietnamese people aimed for, a restless, wandering spirit was the opposite and undesirable. Some participants even emphasised that a wandering spirit with no place to rest would become starved and consequently become a devil.

*P: It is our Vietnamese custom that we mustn’t take the body home if the person dies somewhere else [outside home].*

*I: Why so?*

*P: Because people believe that the person will then become a devil. (A family member in Hanoi)*

##### 3.1.2.2 A good death is having no worries or regrets

Having no worries or regrets for anything in life and having all of their wishes fulfilled was also a critical part of *“a good death”.* This was why it was also important to see people one final time prior to their death.

*A good death means that the person feels at ease. Their heart feels light. They don’t worry about any duties that they had to fulfil in this life. (A community member in HCMC)*

From another angle, “*a good death”* for many Vietnamese people also meant that the deceased did not see their family suffering during and after their death. Then the deceased would not feel guilty for their departure, which they already knew caused deep grief to their loved ones. Some participants mentioned that they would restrict their emotions so that the spirit of the deceased would not linger around because of the guilt for causing so much pain to the family.

*[A good death] just means resting in peace, as simple as that. To achieve that, their heart must be in peace. They don’t feel guilty that their departure leaves so much pain to others. If [the family] keeps crying and calling them out loud, how could they be in peace? (A family member in Hanoi)*

For the family, accepting the death of a loved one was hard. From the discourse of the participants, it became clear that the family also strove for no worries or regrets meaning that they had done everything they could for the deceased, whether it was taking care of the deceased before death or taking care of the resting place. This would make the family feel more at ease.

*To let them feel peace, let the living be in peace. Those who are gone are gone. But the living can rest assured that … that they have fulfilled their duty. Besides, the living has a duty … to fulfil their duty to the deceased. (A family member in Dak Lak)*

##### 3.1.2.3 A good death follows practices

In Vietnamese culture, *“a good death”* also conveyed how the spirit of the deceased was treated after their death. There were many different practices and rituals for the deceased after they died. For example, particularly in rural areas, a big and loud funeral was very important to satisfy the spirit. It was widely believed that better preparation of the funeral and of the deceased's grave and spirit, the happier the spirit would be. If the family failed to hold a proper funeral, they would be judged by the neighbours, and indeed the deceased, for not fulfilling filial piety (Son & Nga, 2019).

*If an elderly [person] dies, yet there’s no clarinets and drums at the funeral, people will think that our family doesn’t care [about the deceased]. A proper funeral has to have the noise from the clarinets and drums. (A family member in a rural area of Hanoi)*

It is a long and widely practiced tradition in the country, regardless of the ethnic groups that we had the chance to talk to in this study, that the family will visit the dead's resting place, wherever it may be, or burn incense regularly at home in their honour. Therefore, looking at a deeper meaning of such practices, *“a good death”* also meant that the person is remembered by their loved ones, even though a long time has passed.

*We will go visit them like other ethnic groups. We will go to where we bury them, then we will use wet cloth to cleanse. It’s our custom. We do this very frequently. Once a week, every three days, every other day, we do this a lot! (An Ede person in Daklak)*

##### 3.1.2.4 A good death – an intact body

Besides spiritual meanings, another salient point for *“a good death”* relates to the body of the deceased. In Vietnamese culture, damage to the body is often regarded as highly prohibited because any cut, even minimal, can cause pain to the deceased's spirit. The body then must remain intact, in its original state, until the body is buried or cremated. Although cremation is becoming more common in the country, an intact body before all the rituals are completed is highly desirable. In this excerpt below, a medical student discussed his consideration for body donation.

*Well, I think if you ask me whether I and my family would choose to donate our bodies [to medicine], I’d say that I still want [my body] to be intact and that [my body] would be brought to my final resting place. (A medical student in Hanoi)*

However, for some people, an intact body was not always important. There were those who chose to donate their organs or body to science. In contrast to people who insisted on keeping the body intact, the body or organ donors often had different perceptions of the body, which we will discuss in the next section.

#### 3.1.3 Shall we talk about a bad death?

It was more frequent for us to discuss the conceptualisation of a good death, but much fewer discussions surrounded what a bad death would entail. In this section, we describe what was considered as a bad death in Vietnamese culture, which further highlights the most important “criterion” of a good death.

Dying outside the home was mentioned most frequently across the narratives of the participants as a negative practice. In Vietnamese, this is called *“chết đường, chết chợ”*, which is literally translated to English as “dying on the streets, dying in the marketplace”. This is used for any cases of death that happened outside the home, including in health facilities. As explained above, if dying outside the home, the spirit will not be able to return home and will eventually become a wandering spirit who will suffer from hunger and thirst. Besides the potentially evil harm that the wandering spirit can cause to the family, it is also the love of the family who does not want to “see” their loved one to become homeless.

Another spiritual aspect of death was related to the time of the death. While a good death occurs in a good (auspicious) hour, a good day, and even a good year, a bad death happens at a bad time when the family will be negatively impacted. In this following excerpt, a doctor shared his experience when the family asked him to try to prolong the patient's life until “a good hour” to die, according to the family's shaman.

*When I was on my duty at the Emergency Department, a family member called me to ask if I could try to keep the patient alive until past 11pm. He said 11pm was a bad hour that can affect the whole family. It’s quite common in ICU and Emergency Department. (A healthcare worker in Hanoi)*

Lastly, it is not a surprise that a damaged body can also cause a bad death for the deceased. Not just making it difficult for the reincarnation of the spirit, the family might also feel the pain when the body is damaged, which also means that the family cannot fulfil their duty to the dead.

*[People] don’t like incompleteness. That’s why they have the belief that when someone dies, the body has to be intact, like if the patient has a brain surgery, they will also request for the removed section to be returned so the body will be complete. (A healthcare worker in HCMC)*

### 3.2 Perceptions of the body

In the previous section, we have described a common belief that to have “*a good death”*, the deceased's body must remain intact, without any damage. This implies that the body bears certain meanings in Vietnamese culture that are worth further exploration in order to more fully understand the meanings of death and dying. This concept is of particular importance in the context of autopsy. From the narratives of the participants, we generated two major themes. We will discuss one perception that was held by fewer participants (i.e. mostly among organ or body donors) that the body was temporary, before describing the more prevalent overarching theme of the sacred body.

#### 3.2.1 The body as temporary

As briefly mentioned in the previous section, some people believed that the body would be gone, therefore, there was not a need to protect it. From the perspectives of some religions, it was the spirit that was the most important part of a human being. When a person dies, the spirit would travel in a new journey, while the body will be gone. The body, therefore, was just a temporary home for the spirit in this life.

*This body … we have to understand it as a boat that takes us to the other side. It’s not that precious that we need to protect it all the time. Because it’s temporary, how can we keep it forever? It’s not eternal. (A community member in HCMC)*

Such beliefs were particularly common among those who registered for organ or body donation. Beyond the impact of their religions, these people believed that since the body would be gone anyway, they wanted to contribute their bodies to help others. The people who believed in the temporary body were more likely to be in favour of MITS.

*I think the body can be helpful after death. You see, the body will be buried or cremated anyway, there’s no meaning in that. Speaking in a raw way, it’s like the body is thrown away, nothing left behind. We have to do something meaningful for this life. (A community member in HCMC)*

#### 3.2.2 The sacred body

The body as sacred was the more prominent perception surrounding the body across the discourse of the participants. In one way, the body as sacred was related to spirituality, that is one of the criteria for “*a good death”*. Further, when someone dies, the body should be kept intact. Damage to the body, whether it was a major cut, or a tiny needle inserted to the body as in MITS, were highly undesirable. This belief was common, even for people with religions that state that the spirit is more important than the body after death. We discussed this with a religious leader whose religious beliefs emphasised the importance of the spirit, not of the body. However, he showed reluctance when we discussed MITS and how it might be accepted by their religious communities.

*They don’t want that, they don’t want their loved one’s body to be cut open. Of course, the body will be returned to them after, they know. But they want the body of their loved one to be intact when burying, then it will disappear naturally on its own. (A community member in HCMC)*

On the other hand, the body was sacred to the family because it represented the person who passed away. In other words, the body is what remains of their loved one. The body was, therefore, respected and looked after by the family and other people after death. This was particularly related to the perception that a good death meant having no regrets, from the family's perspectives. Taking good care of the deceased's body was the last thing they could do to the deceased before saying goodbye. Therefore, various preparations for the funeral always included cleansing the body thoroughly and presenting the body in the nicest clothes. This also applied in clinical settings, where healthcare workers would help the family take care of the body in moments of grief.

*Some bodies look fine, like they are just sleeping. But some aren’t. In such cases, we will cleanse them thoroughly. We will change clothes for them so that they will feel most comfortable. Some men in my village do it very well, you know. They do it while talking to the deceased, like “Let me move your hand a bit” or “Will you lie down here? I will fix your leg”. (A family member in Hanoi)*

## 4 Discussion

In this paper, we explored the perceptions of death and dying, with particular focuses on the meanings of *“a good death”* and of the body in Vietnamese culture. We found that the perceptions surrounding death were multifaceted and might not always align with how people chose to face death. While some people chose to prepare for themselves and their loved ones in consideration that death was inevitable, others avoided talking about it and only surrendered when there was nothing more they could do. Central to the perceptions of death, “*a good death”* was a core belief that everyone wanted to obtain in different ways. To have no regrets or worries was an overarching theme for both the deceased and their family. The body was seen as sacred, because it was the remains of the deceased, therefore, it should be cared for, protected and respected by the family and others. Our study did not find any clear differences in the perceptions comparing health workers with the general public.

We found that among various perceptions of death, most participants recognised it as an inevitable event where an individual's journey in this life comes to an end. This perception is not rare in Asian countries on which Confucianism used to have profound influences (Lee, 2009; Sallnow et al., 2022). The attitudes toward death, however, might not match this perception (Son & Nga, 2019). On one hand, an attitude of acceptance, as well as the physical and emotional preparations for their departure as narrated by some participants were echoed in a previous study in Vietnam that explored attitudes towards death among cancer patients in Vietnam where the patients chose to accept their fate (Long et al., 2018). Similar attitude to death, that is *“let death take its course”* - was also found in a qualitative study conducted in China whose culture share some common characteristics with that of Vietnam (Lei et al., 2022). On the other hand, not everyone had such a calm approach to facing death, especially when death seemed to come nearer. The fear of death, as demonstrated by some people in this study, has been frequently described in literature on death, in both Western and Eastern cultures. In Vietnamese culture, the fear of death is indicated through the avoidance of mentioning death that could be due to the fear of losing a loved one or, for a dying person, the fear of transforming into nothing as opposed to what would happen within their belief system (Son & Nga, 2019). The denial of talking about death was also discussed by Lei et al. (2022) in the aforementioned paper where the authors observed that death was rarely mentioned in everyday life among the elderly and even in the family for the fear of bad luck or the children not wanting their parents to worry about the unknown (Lei et al., 2022). In Western culture, death anxiety has been widely discussed in different theories or frameworks where some scholars proposed that the fear of death might be due to the regret for this life (Sallnow et al., 2022). Click or tap here to enter text. For family members of the dying patient, the anxiety might come from separation anxiety where they fear of losing an attachment figure (Iverach et al., 2014). Thus, the talk about death can never be easy with myriad evidence from previous studies (L. T. Nguyen et al., 2014; Sallnow et al., 2022; The, 2001; Tsao et al., 2019). While attitudes toward death vary across cultures and even within a country, the findings from this study further underlined the importance of communication about death and emotional support to ensure that community members will not be evoked with any painful feelings or irritation.

As mentioned above, central to any discussions about death and dying is the perception regarding *“a good death”*. Current literature recognises that “*a good death”* is a multi-layered concept that can be perceived in various ways in different cultures (Sallnow et al., 2022), however, dying at home with the presence of loved ones has been emerging as one of the most crucial criterion for a beautiful departure (Zaman et al., 2021). Research from an ethnography study conducted in Intensive Care Units in Vietnamese hospitals also found that it was common for families to take the patient home when the prognosis was moribund (*forthcoming*). In Vietnamese and other Asian cultures, dying at home with the company from loved ones is the first and foremost criterion of “*a good death’*, for both emotional, moral and spiritual reasons (C. Chen et al., 2022; Cheng et al., 2025; Keratichewanun et al., 2023; Son & Nga, 2019; S. D. Stonington, 2012; Wijeyaratne et al., 2025). As Stonington (2012) put it: *“The home is sacred and familiar. It is familiar and warm”* (S. D. Stonington, 2012, p. 43). Emotional support and care from family members are indeed imperative for the dying person, but at the same time, in some cultures such as Vietnam, Thailand and China, taking care of your parent until the last moment is an act of filial piety – a moral value rooted in people's belief (Cheng et al., 2025; Son & Nga, 2019; S. Stonington, 2020). In Western cultures, which recently faced increasing criticism for the medicalisation of the dying experience (Zaman et al., 2021), there are different perspectives for a “good” dying place. In recent years, there has been a shift where home has become more common as dying patients expressed their wish to be able to die at home, emphasising the importance of a familiar environment to an individual in their last days (Cottrell & Duggleby, 2016; Zaman et al., 2021). This was also the overarching theme in a project exploring experiences of dying at home for patients living in deprivation in Scotland where researchers found that despite the costs and poor conditions, the home environment was still where they wanted to remain for a long time (Richards et al., 2024). Despite this, some studies highlighted a different choice of terminally ill patients in some Western countries (e.g. Canada, Australia) in choosing their dying place other than home. This was particularly the case for those who did not have close relationships with their families. Some expressed the comfort of being at home at their last days, but worried to burden their families and other complexities of medical care emerged if staying home (Etkind et al., 2018; Evans et al., 2006; Funk et al., 2022; MacArtney et al., 2016). While this differs from our findings, it suggests an interesting avenue to explore further for end-of-life care research in Vietnam and other countries of similar cultures.

In the Results, we described a common belief in Vietnamese culture that dying outside the home will result in a wandering spirit, with nowhere to shelter, who become unhappy and evil, and return to bother the family (Bodemer, 2005). The deceased and their family will then not be able to be in peace. This is also the spiritual meaning of dying at home in Vietnamese culture. The scene where the family rushed to transport the patient home at all costs, which was termed as “choreographing” a good death, was also narrated in The Spirit Ambulance, where the author described and discussed the socio-cultural and even spiritual implications behind end-of-life decision making in Thailand (S. Stonington, 2020). Similar to Vietnam, many people in Thailand also decided to transport the dying person home, believing that there was no other perfect place like home for the person to have *“an effective and safe rebirth”* (Stonington, 2020, p. 23). Due to this popular belief, any interventions concerning death such as MITS might face challenges in terms of recruiting participants or even violate the core perceptions related to death and dying in the local cultures. On the other hand, this suggests the need for end-of-life care to consider practices at home to ensure that they align with Vietnamese cultures and beliefs.

Instead of an individual affair as in most Western cultures (Kehl, 2006), *“a good death”* in Vietnamese culture was portrayed more as a communal one where the deceased's concerns about family and the substantial involvement of family in a person's death shaped the conceptualisation of *“a good death”*. First, our finding where family was deeply involved in the dying process of the person was similar to a study in China where the authors found that children tried to prolong parent's life as an act of filial piety (Cheng et al., 2025). We also found that despite different cultures and beliefs, a common theme of *“a good death”* in any country is no worries and not burdening the family after death (Cheng et al., 2025; Cottrell & Duggleby, 2016; Fu & Glasdam, 2022; Keratichewanun et al., 2023; Wijeyaratne et al., 2025; Zaman et al., 2021). The important position of the family in shaping the dying experience of a person has an important implication that beyond bereavement that the family has to endure as described in current literature (Sallnow et al., 2022), the family also needs support in addition to support for the dying experience of the person. End-of-life care, therefore, is necessary to involve the whole family, instead of focussing on providing clinical and emotional care for terminally ill patients.

Another key finding of this study was the various views about the meaning of the body in Vietnamese culture. The participants' perceptions were heterogeneity, from strict prohibition on the damages on the body to the perception that the body was temporary, however, the denial of any damages to the deceased's body was a more dominant view. This finding aligns with the results from previous studies on body donations in other Asian countries where the authors observed that although the perceptions of body donation had changed, people, especially the elderly, more often preferred to keep the body intact due to the influences of Confucianism (W.-L. Chen, 2023; Zhang et al., 2020). In our study, the impacts of Confucianist views were not explicit, however, we found that many participants believed that an intact body was critical to have “*a good death”*, which was important to achieve. Despite distinct views on the body after death, as described in the results, the body had a sacred position as illustrated by how people cherished the body or what remained of it as being physically not with them anymore (Schwartz, 2015). This has important implications for any related practices, such as MITS and body donation, that respect for the body is key to obtain consent and improve acceptability from the general public.

### 4.1 Implications for MITS

Understanding the socio-cultural views surrounding death and the body will not just help us better shape the implementation of MITS to be more culturally appropriate, but, thinking more broadly, it also has implications for other relevant procedures, such as organ and body donation.

Based on these findings, we identified several spaces where MITS could be influenced. First, while the home was the ideal place where a dying person wished to spend their last days, it could restrict the practice of MITS, which is mostly conducted in health facilities. Second, family involvement in MITS implementation is critical. Transparent communication as well as following ethical procedures for consent are key to gain trust from the family. This is not just to increase the rate of consent to MITS, but also to ensure a good death situation for the surviving family. Finally, understanding how important the body is for people is a crucial consideration for post-death medical procedures, whether MITS or body/organ donation, treating the body or the remains with respect is the main responsibility. For MITS, all damage on the body should be kept minimal, and it should be thoroughly cleansed before returning it to the family. Similarly, for body and organ donation, when the body is with the medical staff, it should be treated with great care and respect before returning the body back to the family. This is first to respect the deceased and the family, while more broadly reflecting appreciation for the local cultures and customs (Suwalowska et al., 2023).

### 4.2 Limitations and strengths

Our study has several limitations and strengths.

First of all, despite efforts, we could only interview two participants of an ethnic minority group (Ede), which made our descriptions of death and dying provided in this article unable to illustrate the overarching themes among the other ethnic groups across Vietnam impacting the potential transferability of findings to these groups. As the main purpose of the study was to explore the hypothetical acceptability of MITS, not to solely examine the perceptions of death, dying and the body, ones who were looking for more in-depth ethnographic descriptions might be disappointed. The results of this study will benefit from further complementary studies exploring death and dying in different ethnic groups’ cultures to enable exploring any implications for related practices. Finally, we did emphasize gender as an analytical lens, and therefore may have inadvertently lumped together perceptions and practices that might be gender specific.

However, our study also had many strengths. Although published literature on death and dying in Vietnam is not rare, to our knowledge, this is one of few studies exploring the conceptualisation of *“a good death”* and the meaning of the body in Vietnamese culture. This paper will contribute to the emerging literature on death and dying in Asian cultures in the recent years in the situation where studies on this topic from Western countries has been more dominant. Another strength is that we further underlined the importance of end-of-life care in Vietnam, where such practices are now in high demand (V. Nguyen et al., 2023). This also aligns with the recognition of the salience of this issue with the establishment of the Lancet Commission on death (Sallnow et al., 2022).

## 5 Conclusion

In this paper, we explored the multifaceted perceptions of death and dying and the meaning of the body after death in communities in Vietnam. Death and a good death in Vietnam are more of a communal event, rather than an individual affair. These findings demonstrate important implications when thinking about promoting MITS, in terms of shaping the procedures to maintain respect for the deceased, their body and the family and wider communities. If MITS is ever implemented in Vietnam in the furture, ensuring an understanding of these perceptions and practices will improve its acceptability.

## CRediT authorship contribution statement

**Nhung Phuong Doan:** Writing – review & editing, Writing – original draft, Investigation, Formal analysis. **My Nguyen Le Thao:** Writing – review & editing, Investigation. **An Luu Phuoc:** Writing – review & editing, Investigation. **Alix Day:** Writing – review & editing. **Halina Suwalowska:** Writing – review & editing, Conceptualization. **Ho Dang Trung Nghia:** Writing – review & editing, Supervision. **Mary Chambers:** Writing – review & editing, Supervision, Conceptualization. **Ta Thi Dieu Ngan:** Writing – review & editing, Supervision, Funding acquisition, Conceptualization. **H. Rogier van Doorn:** Writing – review & editing, Supervision, Funding acquisition, Conceptualization. **Jennifer Ilo Van Nuil:** Writing – review & editing, Supervision, Formal analysis.

## Funding sources

This research was funded by the Viet Nam Call in Infectious Diseases, Medical Research Council Newton Fund UK (MR/R0263001/1 to RvD) and Ministry of Science and Technology in Viet Nam (NDT.78.UK/20 to NTTD): Establishing and evaluating minimally invasive autopsy to determine cause of death from infectious disease in Viet Nam. The funders played no role in the design, data collection, analysis, or write-up of this research.

## Declaration of competing interest

The authors declare that they have no known competing financial interests or personal relationships that could have appeared to influence the work reported in this paper.

## Footnotes

2 Dak Lak was the province during data collection, however, in 2025, Vietnam reorganised the administrative boundaries across the country and therefore this province was merged with a neighbour province, while keeping the original name Dak Lak for the new merged province. Dak Lak province, which was mentioned in this manuscript, refers to the old one.
