# Supplementary material for: Perceptions of death, dying and the body in Vietnamese culture: A qualitative exploration from a study examining the potentials of minimally invasive tissue sampling in Vietnam
Source: SSM Qual Res Health. 2026 Jun;9:100714. doi: 10.1016/j.ssmqr.2026.100714 (PMC13328329; doi:10.1016/j.ssmqr.2026.100714)
Supplement: Multimedia component 1 [file mmc1.docx]

# APPENDIX. Data collection tools

1. **Interview guide for community stakeholders**

**Community** *(members of the public, professionals involved in proceedings related to death and dying, knowledgeable people in the community, community level health professionals).*

**Opening**

1. Tell me a little more about yourself and [*if applicable*] your current role at work?

**THEME I: Death and related practices**

1. Please describe what happens when a person dies in your community.

*Probes*:

- What cultural and/or religious practices and rituals (all rituals from when the person dies) will be conducted?
- Have the practices/rituals changed overtime? Why?
- Who will usually participate in these rituals?
- What happens to the corpse?
- Would the practices/rituals be the same for everyone? For e.g.: when a child dies or when an elderly dies
- How and when does the family tell the community that a person/child has died?
- How would community members/neighbours support the family?

1. Will there be any private ceremonies or rituals conducted in the family? If yes, please elaborate.

- What are the meanings of these events?
- Who usually conducts these? Who needs to be present?

1. What are the burial procedures?

- Are there variations in the practices in Vietnam? If yes, please elaborate.
- What is the cost of burial procedure? Who covers the costs?
- Do all family members participate?
- Is there any ritual conducted after the burial procedure? If yes, please elaborate.

1. Is anything done long after a person has died (e.g., at the anniversary of the death)? If yes, please elaborate*.*
2. What are the beliefs around death and the deceased’s body in your community/culture/religion?

*Probes:*

- What is the concept of a good death?
- What does the deceased’s body mean to the family?

1. Do you feel there is value in knowing the cause of death of a person? Why?

*Probes:*

- Explore the desire/willingness to consent.
- How much information would be valued?
- Who should give this information to the family?

**THEME II: Autopsy**

**[*provide brief description of autopsy if necessary*]**

1. What have you heard about the autopsy procedure?

*Probes:*

- What is the purpose of that?
- When is the autopsy procedure requested?
- Who requests it?
- Where is it conducted?
- Who conducts it?
- Do families have a right to refuse the procedure?

1. Have you heard about any cases of autopsy in your community/family?

*If yes, probes:*

- Please describe the situation.
- What were the circumstances leading to the autopsy?
- Who requested the autopsy? Who conducted the procedure?
- What was the reaction of the family members and the community?
- What happened after the autopsy?

1. What is the general perception about autopsy in your community?

*Probes:*

- What is your view about this? What about others’?
- What are the concerns around it?
- Why do you think people have such perception and concerns about autopsy?
- What people may think about touching/cutting the dead body in your cultural/religious/ethical context?
- Is it different for children, elderly, adults, pregnant women, etc.?

1. What is your perception of people who are involved in the procedure? What other people in the community may think about them?
2. In your view, what is the purpose of an autopsy? What are the benefits to the families, if any, to consent to the autopsy procedure?
3. How should a family decide about autopsy?

*Probes:*

- Is that a family decision or an individual decision/who decides?
- How might the community react when a family decides to consent to an autopsy?

**THEME III: Minimally invasive tissue sampling**

***[Explain the procedure.]***

1. What are your initial thoughts about the procedure?

*Probes:*

- What would be the perceived barriers/objections against conducting MITS? How might these be overcome?
- Are there any concerns about cultural/ethical/religious issues and the delay of burial plan?
- What would be the perceived benefits for conducting MITS?
- What would the community think about this method?

1. What are the circumstances under which the MITS procedure would be acceptable?

*Probes:*

- Would MITS be acceptable in different age groups (children, elders, pregnant women, adults)?

1. How could MITS be explained to family members?

*Probes:*

- Who should give consent to MITS?
- Who should explain the process to the family and acquire consent?
- What information should be explained to the family?
- Should any kind of incentive/compensation be given to the family? If yes, what should it be?
- Should the results be given to the family? If yes, how and what information should be given?

1. How should MITS be adapted?

*Probes:*

- Where would MITS be ideally performed?
- When should the procedure be conducted?

**Closing**

1. Are there any additional comments you would like to add to the discussion?

**END OF THE INTERVIEW**

1. **Interview guide for family members**

**Family members** *with relatively recent experience of involvement in discussions about autopsy and/or cause of death.*

**Opening**

[Build rapports with the participant about their family, occupation, etc.)

1. Please tell me how your *(wife, brother, sister…)* died?

*Probes:*

- How long have they passed away?
- Could you share why the person died?

**PERCEPTION OF DEATH, DEAD BODIES AND TRADITIONAL RITUALS**

1. What rituals and practices were conducted?

*Probes:*

- What happened to the deceased’s body?
- Rituals and practices after the person died until the burial procedure was completed
- Rituals and practices after the person died for some time
- Who participated in these rituals/procedures/practices?
- Do you know the meanings of these rituals or practices? If yes, please elaborate.

1. Were the rituals performed similarly in your community? If not, please describe how your family’s rituals differ and why.

*Probes*:

- Does your community have any distinctive rituals or practices related to death and dead bodies? If yes, please elaborate.
- How would the community members support your family?

1. What is the perception of a good death?

*Probes:*

- In your perspective, what is a good death? Does the body of your beloved mean anything to you and your family?
- What is the perception of a good death in your community?

1. When your family member died, did you and your family want to know the cause of their death? Why/Why not?

Probes:

- How was the CoD determined?
- What do you and your family think about the given CoD?

**COMPLETE DIAGNOSTIC AUTOPSY**

1. Was an autopsy performed to determine your family member’s CoD?
2. Have you been aware of an autopsy performed in your community?

*If the participant answers Yes to one of the questions above, continue asking the following questions:*

1. Please describe what happened related to the autopsy:

*Probes:*

- Who requested the autopsy?
- Was the purpose of the procedure explained to you? If yes, what was its purpose?
- How long after the person died was the autopsy procedure conducted?
- Where was it conducted? Who conducted it?
- Were there any concerns? If yes, how were the concerns addressed?
- How quickly the body was released?
- Have any form of compensation (*transport, coffin etc.)* be given*?*
- Were the family informed about the result of the autopsy?

1. Was your/the family asked for consent?

*Probe (if yes):*

- Who asked for consent?
- What information was provided?
- How was the decision made? Who made the decision?
- Why did your/the family agree/disagree?
- Did you consult anyone to make this decision? (or it was solely family decision)

*General questions about CDA to all participants:*

1. What is your view about the autopsy?

*Probes:*

- Are there any socio-cultural, religions or ethical issues related to autopsy? Please elaborate.
- What do you think about the health workers who perform autopsy?
- In what circumstances should an autopsy be conducted? Why so?
- In what circumstances should an autopsy NOT be conducted? Why so?

1. What do you think other people in your community think about the autopsy procedure?

- What are the beliefs about what should/should not happen after a person dies?
- How does the autopsy procedure fit with those beliefs?
- What do you think might be the concerns of people in your community related to autopsy?
- Do you think families should have the right to refuse autopsy? Why or why not?

**Minimally invasive tissue sampling**

**[Explain the procedure.]**

1. What are your initial thoughts about the procedure?

*Probes:*

- Would MITS be more acceptable in your community? Why?
- Would there be any socio-cultural, religious and ethical issues to implement MITS? If yes, please elaborate.
- What would be the perceived barriers/objections against conducting MITS? How might these barriers be overcome?
- What would be the perceived benefits for conducting MITS?

1. Would you give consent to MITS to determine CoD of your family member? Why?

*If yes, probes:*

- What information about MITS should be explained to you and your family? In what methods?
- Who would you like to seek consultation before consenting?
- Who should approach the family for consent and when? and how?
- Where would MITS ideally be performed?
- Should incentive/compensation be given? If yes, what kind of incentive/compensation?

1. What might your community think about MITS?

**Closing**

1. Are there any additional comments you would like to add to our discussion?

**END OF THE INTERVIEW**

1. **Interview guide for healthcare professionals**

**Opening**

General experience

1. What would you describe as your main role at work?
2. Have you been involved in much work involving autopsy procedures? In what capacity?

**THEME I: Procedure for death**

1. Please describe what happens when a person dies in your hospital?

*Probes:*

- What happens to the corpse?
- Is the procedure the same for every person? If not, please tell me more about how the procedure might differ.
- What is the process for reporting death in your hospital?
- Who is typically involved in the procedure when a person dies in the hospital?
- Are there any legal requirements? If yes, please describe the legal procedure.
- Are there any rituals that are conducted by staff when a person dies? If yes, please elaborate.

1. What is your experience of communicating death and related procedure to the family members?

*Probes:*

- What information is given?
- Who provides the information to the family?
- How is this information typically provided?

1. What are the various beliefs and attitudes regarding death and dead bodies in your community?
   - How do they influence the current procedure for death in your facility?
2. Would the Cause of Death (CoD) be determined when a person dies in the hospital?

*Probes:*

- How would the CoD be determined?
- Who certifies the CoD?
- What information is given?
- In your opinion, what is the validity of that information?
- Are there any legal requirements after CoD is determined? If yes, please tell me what legal procedures need to be done.
- Do you communicate with the family members about the deceased’s CoD? If yes, how?

1. What is your thought about determining CoD?

*Probes:*

- Do you think there is value in knowing CoD? If yes, what benefits can it have?
- Do you think there is a value for families to know CoD? If yes, what benefits would the families have?

**THEME II: Autopsies**

1. Are you aware of any autopsy requested in your hospital?

If yes, ask the following questions:

1. Please explain what is the process of requesting an autopsy procedure in your hospital?

*Probes:*

- Who requests the autopsy procedure?
- When is it requested? (clinical, forensic, for research purposes)
- Do you have post mortem facilities in the hospital? Where are they based?
- How many people work in the mortuary?
- Who conducts the autopsy procedure? How often are they done?
- What training is provided in order to conduct autopsy procedures?

1. What is the value of conducting autopsy procedure in your clinical practice?

*Probes:*

- What are the challenges?
- Do you (or anyone else) have any objections? If yes, why?
- Should there be more or less autopsies conducted? Why?
- What do you think about conducting the complete autopsy in Vietnam? (in relation to socio-cultural, religious, etc. contexts)

1. What have been the family reactions to the autopsy procedures conducted in your hospital?

*Probes:*

- Would the family’s consent be sought before conducting autopsy? If yes, what information would be provided to them? How? Who would do this?
- What are concerns expressed by the family members?
- What is the value of the procedure to the families?
- How much information would be valued by the families regarding CoD?

**THEME III: Cause of death and Minimally Invasive Tissue Sampling (MITS)**

1. What do you know about the MITS?

*(Probe: if no knowledge about MITS information will be provided)*

1. What is your perception about MITS?

*Probes:*

- Do you think MITS is valuable in determining CoD?
- Do you think MITS might have more advantages than the complete autopsy? If yes, in what ways? If no, why?
- What do you think about conducting MITS in the social, cultural and religious context of Vietnam?
- What might be the challenges? How can these be overcome?

1. What do you think about introducing MITS as a method to determine CoD?

*Probes:*

- Where should the MITS procedure be performed? Why?
- What should the timeline be? (e.g.: how long after the patient’s death)

*If the participant answers MITS should be conducted in the hospital, ask:*

- What would the value of introducing MITS in the hospital be?
- What would the reaction from healthcare professionals be?
- What challenges there would be when introducing MITS in the hospital? How might these be overcome?
- What would be the requirements for the hospital to conduct Minimally Invasive Autopsy procedure in terms of administration, training, facilities, staff, etc.?

*If the participant answers MITS should be conducted somewhere else like at the morgue or at the patient’s home, ask:*

- What might the challenges to conduct the procedure here be? How could these be overcome? *(Also ask about the potentially culturally and ethical barriers)*
- What would the reaction from the family and the community be?
- What would be the requirements to conduct MITS outside of a health facility?

1. In your view, how would the family think about MITS?

*Probes:*

- Would it be more acceptable for the family in comparison to the complete autopsy method? Why? Why not?
- How do you think the consent procedure should be done?
  - Who should ask for consent?
  - When it should be taken and from who?
  - What information should be given to the family?
  - Do you think families should be offered something for agreeing to MIA? If yes, why? What could they be offered?
  - What are some strategies to talk to families about MITS?
- Should the result of MITS be informed to the family? If yes:
  - What information should be given?
  - Who would deliver the information?
- What could be done to improve the acceptability of MITS to the families?

**Closing**

1. Are there any additional comments you would like to add to the discussion?

**END OF THE INTERVIEW**

1. **Interview guide for key informants**

**Key informants** (*policy makers from the health, legal, vital registration, public health professionals)*

**Opening**

1. **General experience**
2. What would you describe as your main role at work?
3. Have you been involved in work on measuring mortality and determining Cause of Death (CoD) in Vietnam? In what capacity?
4. How long ago did you get involved? What led you to get involved?
5. How does this fit into your wider work?
6. **Measuring mortality and CoD**
7. How is the mortality and CoD statistics data collected in Vietnam?

*Probe:*

- *How mortality surveillance is conducted?*
- *What are the procedures?*
- *Which institutions are involved?*

1. What is the value of collecting mortality and CoD statistics?

*Probe:*

- *How the data is being used?*
- *Do you think it has informed any policy decisions? If yes, how? If not, why?*

1. What are the methods to determine CoD that have been being used Vietnam that you are aware of?

*Probe:*

- *What is your view on these methods and their reliability? What has informed this view?*
- *What method has been used more frequently? Why?*

1. What are your views on complete diagnostic autopsy (CDA) as a method to determine CoD in Vietnam?

[Explanation about CDA might be given here, if necessary]

*Probe:*

- *How often is the method used?*
- *In what circumstances?*
- *What are the values of CDA?*
- *What have been the challenges?*

1. **Ideas around MITS**
2. Are you aware of Minimally Invasive Tissue Sampling (MITS/MIA) as a method to determine CoD?

[Explain the procedure if the informants are not familiar with it]

1. What are your views on MITS?

*[Probe: What has informed this view?]*

1. What are your views on the value of MIA in mortality surveillance?

*Probe:*

- *How useful is MIA in* clinical management*?*
- *How useful is MIA in your specific work/research context?*
- *What is the role of MIA in providing diagnosis about non-communicable diseases, for example?]*

1. Do you see introducing MIA as more acceptable to determine CoD in Vietnam that you identified earlier?

*[Probe: If yes, what has informed this view? If no, why not? ]*

1. What are the opportunities of introducing MIA in Vietnam?

*[Probe: What has informed this view? What are the facilitators of introducting MIA in Vietnam?]*

1. What are the barriers of introducing MIA in Vietnam?

*Probe:*

- *Why do you think those are the barriers?*
- *What is different between Vietnam context’s barriers and other context’s?*
- *Do you think these bariers can be overcome? If yes, how? If no, why not?*

1. What can be done to improve the acceptability of MIA in Vietnamese context?

*[Probe: what to do with policy/education/communication etc.]*

1. In the long-term, do you feel that MIA stands a strong change of becoming a mainstream tool in your context?

*Probe:*

- *If yes, why? if no, why not?*
- *In your opinon, what will happen if MIA finally becomes a mainstream tool? what can be the reactions from HCW and community?*

1. Do you think that there are any other alternative methods which are preferable to MIA? *[Probe: What has informed this view?]*
2. **The future of measuring mortality and CoD in LMICs and in Vietnam**
3. To your knowledge what are the challenges to effective measuring of mortality and CoD in low and middle income countries (LMICs) and in Vietnam?

*Probe: What need to be improved?*

1. How do you see the future of measuring mortality and CoD in LMICs and in Vietnam?

**Closing**

1. Are there any additional comments you would like to add to our discussion?

**END OF THE INTERVIEW**
